# Enhanced multi-class pathology lesion detection in gastric neoplasms using deep learning-based approach and validation

**DOI:** 10.1038/s41598-024-62494-1

**Published:** 2024-05-21

**Authors:** Byeong Soo Kim, Bokyung Kim, Minwoo Cho, Hyunsoo Chung, Ji Kon Ryu, Sungwan Kim

**Affiliations:** 1 Interdisciplinary Program in Bioengineering, Graduate School, Seoul National University, Seoul, 08826 Korea; 2 Division of Gastroenterology, Department of Internal Medicine, Seoul Metropolitan Government Seoul National University Boramae Medical Center, Seoul, 07061 Korea; 3 Transdisciplinary Department of Medicine, Seoul National University Hospital, Seoul, 03080 Korea; 4 Department of Internal Medicine and Liver Research Institute, Seoul National University Hospital, Seoul National University College of Medicine, Seoul, 03080 Korea; 5 Department of Biomedical Engineering, Seoul National University College of Medicine, Seoul, 03080 Korea; 6 Artificial Intelligence Institute, Seoul National University, Seoul, 08826 Korea

**Keywords:** Endoscopy, Malignancy, Gastric cancer, Convolutional neural networks, Colonoscopy, Gastrointestinal diseases, Biomedical engineering

## Abstract

This study developed a new convolutional neural network model to detect and classify gastric lesions as malignant, premalignant, and benign. We used 10,181 white-light endoscopy images from 2606 patients in an 8:1:1 ratio. Lesions were categorized as early gastric cancer (EGC), advanced gastric cancer (AGC), gastric dysplasia, benign gastric ulcer (BGU), benign polyp, and benign erosion. We assessed the lesion detection and classification model using six-class, cancer versus non-cancer, and neoplasm versus non-neoplasm categories, as well as T-stage estimation in cancer lesions (T1, T2-T4). The lesion detection rate was 95.22% (219/230 patients) on a per-patient basis: 100% for EGC, 97.22% for AGC, 96.49% for dysplasia, 75.00% for BGU, 97.22% for benign polyps, and 80.49% for benign erosion. The six-class category exhibited an accuracy of 73.43%, sensitivity of 80.90%, specificity of 83.32%, positive predictive value (PPV) of 73.68%, and negative predictive value (NPV) of 88.53%. The sensitivity and NPV were 78.62% and 88.57% for the cancer versus non-cancer category, and 83.26% and 89.80% for the neoplasm versus non-neoplasm category, respectively. The T stage estimation model achieved an accuracy of 85.17%, sensitivity of 88.68%, specificity of 79.81%, PPV of 87.04%, and NPV of 82.18%. The novel CNN-based model remarkably detected and classified malignant, premalignant, and benign gastric lesions and accurately estimated gastric cancer T-stages.

## Introduction

Upper gastrointestinal endoscopy plays a pivotal role in diagnosing and managing upper gastrointestinal disorders^1^, with early identification and precise diagnosis of lesions significantly affecting treatment strategies and overall prognosis^2^. Countries with a high prevalence of gastric cancer that have implemented national screening programs with upper gastrointestinal endoscopy have experienced an increase in the early gastric cancer detection rate and a decrease in gastric cancer mortality^3–5^. The early detection and precise diagnosis of gastric lesions, particularly malignant and premalignant lesions, are essential for timely and effective treatment, ultimately leading to enhanced survival rates^6,7^. In addition, when malignancy is suspected, endoscopic prediction of invasion depth is important for deciding treatment modalities, such as endoscopic resection and surgery^8^.

However, the accurate identification and diagnosis of gastric lesions during endoscopy requires a thorough inspection of the stomach, discrimination of abnormal lesions from normal gastric mucosa, and the decision to perform a biopsy, which can be influenced by the expertise and experience of endoscopists^9^. Notably, missed gastric cancer during endoscopy is common, with missing rates of up to 12%^10^. Factors that influence the missing rate include endoscopist errors, such as failure of lesion detection, detection of a lesion without performing a biopsy, and follow-up delays^11^. To overcome these limitations, numerous studies have recently been conducted using convolutional neural networks (CNNs)^12–17^ or machine learning methods^18–21^ to improve lesion detection and differentiation rates during endoscopy.

Deep learning algorithms are widely used in various fields owing to their growing clinical relevance in the medical domain. This can assist clinicians in decision-making^22^, enhance lesion detection, and alleviate the fatigue experienced by endoscopists^23–25^.

In this study, we aimed to develop a novel algorithm for the detection and classification of gastric cancer and premalignant and benign gastric lesions that are commonly identified through upper gastrointestinal endoscopy, while predicting the depth of invasion of gastric cancer.

## Material and methods

### Dataset

**Figure 1:**
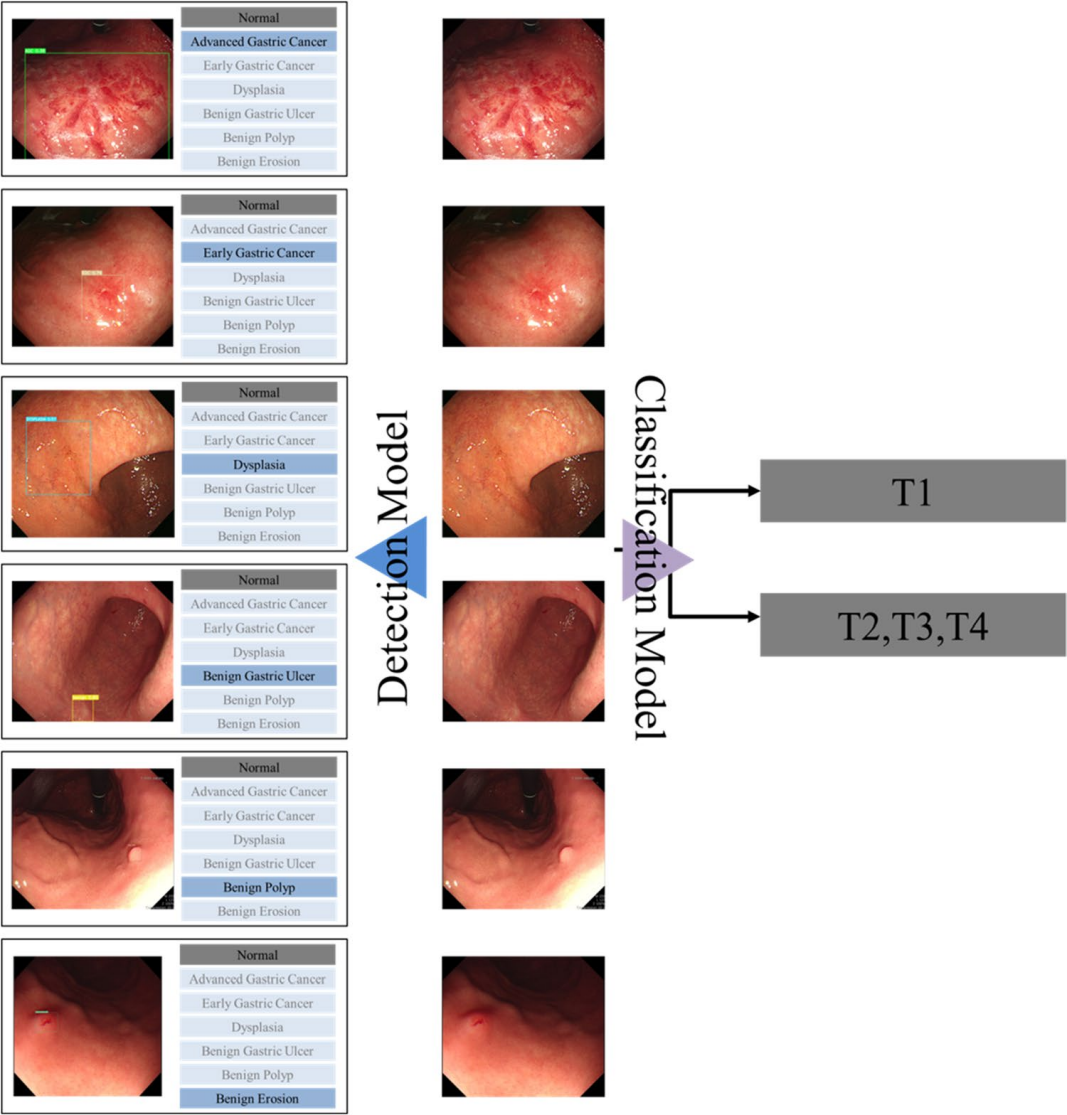
Schematic diagram for the automated Multi-Class Lesion Detection and T-stage Classification Model.

We retrospectively gathered still-image white-light endoscopy images of pathologically confirmed gastric lesions from patients who underwent upper gastrointestinal endoscopy between January 1, 2018, and December 31, 2021, at Seoul National University Hospital (SNUH). These included cases of gastric cancer (early gastric cancer [EGC] and advanced gastric cancer [AGC]), gastric premalignant lesions (low-grade and high-grade dysplasia), benign gastric lesions (benign gastric ulcers [BGU], benign polyps [hyperplastic and fundic gland polyps], and benign erosions), and normal endoscopy cases (normal gastric mucosa with no visible lesions). The exclusion criteria were as follows: (1) inappropriate images (low resolution, blurring, artifacts, bubbles, shadowing, inadequate air inflation, etc.) and (2) images without pathology results (except for images of a normal stomach). The models for lesion detection and invasion depth classification were designed as shown in Fig. 1.

Table 1 shows the composition of image categories in the datasets used in this study. A total of 10,181 white-light images from 2606 participants were included in the study, with an 8:1:1 ratio maintained for the training, validation, and test data to ensure that the patient images did not overlap between the sets. Specifically, the training, validation, and test sets included 1997 (7632 images), 303 (1156 images), and 306 (1393 images) participants, respectively.

**Table 1:** Data distribution of the training, validation, and test sets for the detection model of gastric lesions.

Multi-class detection	Total set		Training set		Validation set		Test set	
	No. of patients (%)	No. of images (%)	No. of patients (%)	No. of images (%)	No. of patients (%)	No. of images (%)	No. of patients (%)	No. of images (%)
Total images	2606 (100%)	10,181 (100%)	1997 (100%)	7632 (100%)	303 (100%)	1156 (100%)	306 (100%)	1393 (100%)
EGC	354 (13.58%)	1398 (13.73%)	284 (14.22%)	1117 (14.64%)	34 (11.22%)	134 (11.59%)	36 (11.76%)	147 (10.55%)
AGC	356 (13.66%)	1405 (13.80%)	285 (14.27%)	1121 (14.69%)	35 (11.55%)	143 (12.37%)	36 (11.76%)	141 (10.12%)
Dysplasia	567 (21.76%)	2108 (20.71%)	454 (22.73%)	1696 (22.22%)	56 (18.48%)	204 (17.65%)	57 (18.63%)	208 (14.93%)
BGU	239 (9.17%)	643 (6.32%)	192 (9.61%)	516 (6.76%)	23 (7.59%)	60 (5.19%)	24 (7.84%)	67 (4.81%)
Benign polyp	353 (13.55%)	915 (8.99%)	284 (14.22%)	737 (9.66%)	33 (10.89%)	86 (7.44%)	36 (11.76%)	92 (6.60%)
Benign erosion	408 (15.66%)	822 (8.07%)	327 (16.37%)	657 (8.61%)	40 (13.20%)	82 (7.09%)	41 (13.40%)	83 (5.96%)
Normal	329 (12.62%)	2890 (28.39%)	171 (8.56%)	1788 (23.43%)	82 (27.06%)	447 (38.67%)	76 (24.84%)	655 (47.02%)

All endoscopic procedures were performed and reviewed by experienced endoscopists, each with more than 6000 cases of prior experience. Gastric cancers and adenomas were treated with either endoscopic submucosal dissection or surgery, and the pathological results of the resected tumors were reviewed.

The lesions were classified by combining endoscopic findings with the pathology reports reviewed by the endoscopists (HSC and BKK). Endoscopic images were classified into six categories: EGC, AGC, gastric dysplasia, BGU, benign polyps, and benign erosions. Images were also classified according to their malignant potential: neoplasm versus benign and cancer versus non-cancer. Cancers included EGC and AGC, whereas neoplasms included both gastric cancer (EGC and AGC) and gastric dysplasia (low-grade dysplasia [LGD] or high-grade dysplasia [HGD]). For gastric cancers, the pathology results of the resected specimens were reviewed, and the depth of invasion was identified as: (mucosal cancer (T1a), submucosal invasion (T1b), proper muscle invasion (T2), subserosal invasion (T3), and serosal invasion or invasion of adjacent structures (T4). The training dataset for the model that classified the depth of invasion is presented in Table 2.

**Table 2:** Data distribution of the training, validation, and test sets for the model classifying the depth of invasion.

T-stage classification	Total set		Training set		Validation set		Test set	
	No. of patients (%)	No. of images (%)	No. of patients (%)	No. of images (%)	No. of patients (%)	No. of images (%)	No. of patients (%)	No. of images (%)
Total images	719 (100%)	2841(100%)	547 (100%)	2167 (100%)	63 (100%)	253 (100%)	70 (100%)	263 (100%)
T1a (mucosal)	279 (38.80%)	1087 (38.26%)	224 (40.95%)	876 (40.42%)	27 (42.86%)	105 (41.50%)	28 (40.00%)	106 (40.30%)
T1b (submucosal)	143 (19.89%)	583 (20.52%)	115 (21.02%)	474 (21.87%)	13 (20.63%)	56 (22.13%)	15 (21.43%)	53 (20.15%)
T2 (proper muscle)	77 (10.71%)	309 (10.88%)	62 (11.33%)	249 (11.49%)	7 (11.11%)	29 (11.46%)	8 (11.43%)	31 (11.79%)
T3 (subserosal)	112 (15.58%)	436 (15.35%)	90 (16.45%)	350 (16.15%)	10 (15.87%)	40 (15.81%)	12 (17.14%)	46 (17.49%)
T4 (serosal exposure)	69 (9.60%)	268 (9.43%)	56 (10.24%)	218 (10.06%)	6 (9.52%)	23 (9.09%)	7 (10.00%)	27 (10.27%)
N/A	39 (5.42%)	158 (5.56%)	–	–	–	–	–	–

### Characteristics of the included images

The data were categorized into six classes, as listed in Table 1. Specifically, 48.24% (4911/10,181) of the entire dataset fell into the “neoplasm” category (including EGC, AGC, HGD, and LGD), 23.38% (2380/10,181) were classified as “non-neoplasm” (which included BGU, benign polyps, and benign erosions), and 28.39% (2890/10,181) as “normal mucosa.”

Within the neoplasm category, dysplasia images comprised the highest proportion at 20.71% (2108/10,181), followed by AGC at 13.80% (1405/10,181) and EGC at 13.73% (1398/10,181). In the non-neoplasm category, benign polyp images constituted the largest portion at 8.99% (915/10,181), followed by erosions at 8.07% (822/10,181), and BGU at 6.32% (643/10,181). Normal mucosa images were not separately categorized during training; however, they were used as background images and negative examples in the test set. Endoscopic images were extracted from the picture archiving and communication system of SNUH in PNG format and captured using Olympus Medical Systems endoscopes (GIF-H290) and video processing systems (EVIS LUCERA ELITE CV-290) in Tokyo, Japan. Furthermore, to anonymize the patient data, sections corresponding to patient information were cropped and removed from the original endoscopic images. Consequently, only the images corresponding to the field of view of the gastrointestinal endoscope were obtained through preprocessing (the minimum resolution of these images was 371 × 322 pixels).

**Figure 2:**
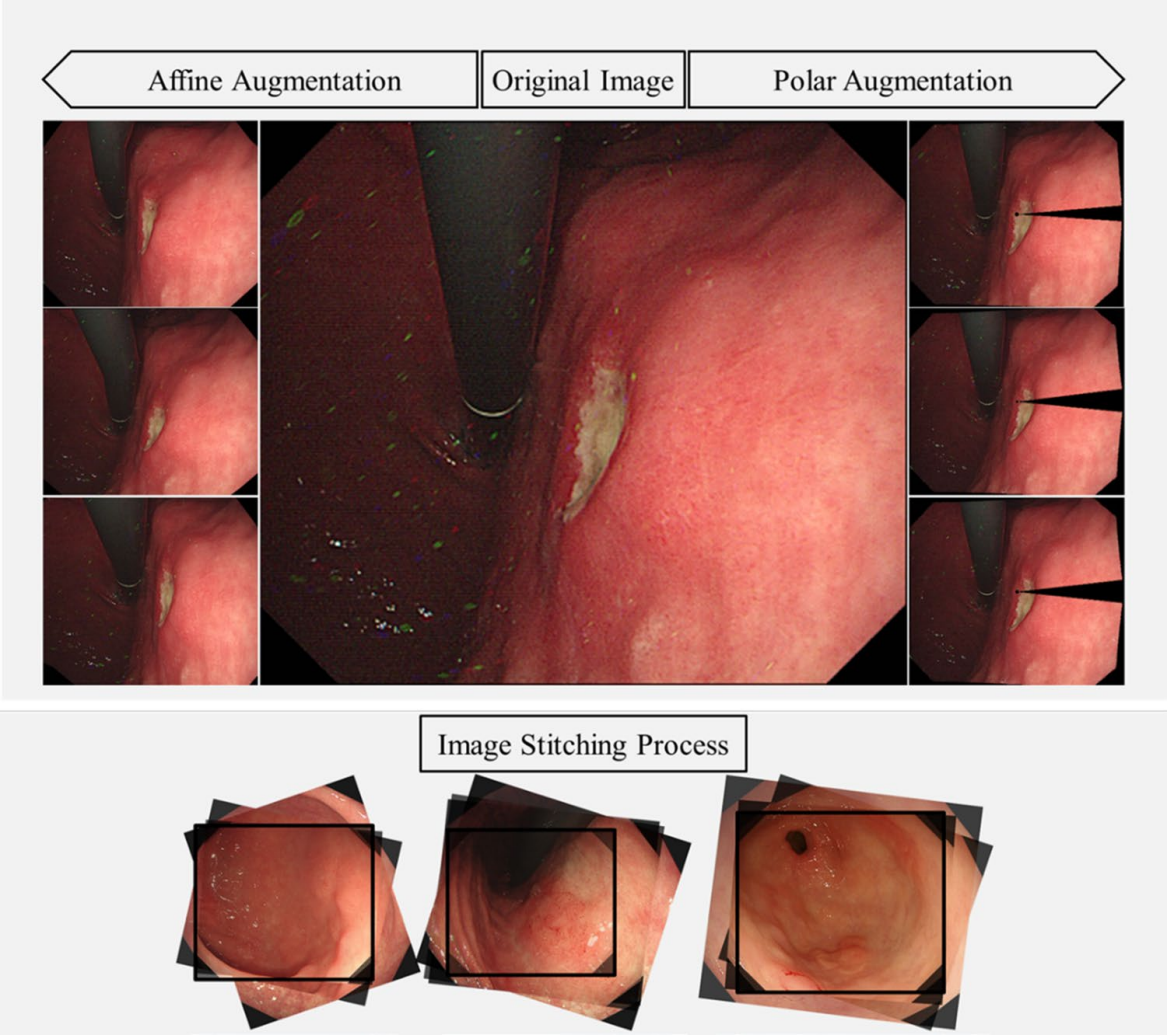
Application examples of image augmentation: augmentation method using imgaug library, involving affine transformations on the left and polar augmentation on the right of the original image. Image stitching with homography aligns multiple multi-angle lesion images for augmentation.

In medical datasets, achieving a natural balance can be challenging, resulting in imbalances in the number of data points across different lesions when the images are used for classification training. Various methods have been used to address this issue. In this study, we adopted data augmentation techniques (Fig. 2), including horizontal flip, HSV channel translation, affine augmentation, polar augmentation^26^, mosaic augmentation^27^, and copy paste augmentation^28^. Lesion images from patients undergoing upper endoscopy typically consist of approximately three to four images per patient, taken from various angles and distances; therefore, we addressed class imbalance by employing image stitching^29^ in the validation set (Fig. 2). The authors assert that all procedures contributing to this work complied with the ethical standards of the relevant national and institutional committees on human experimentation and the Declaration of Helsinki of 1975, as revised in 2008. The requirement for written consent was waived by the Institutional Review Board (IRB) of Seoul National University Hospital (no. 2108-030-1242; the IRB acquisition date of the IRB is August 31, 2021).

### Model development and main outcome

All deep learning models were developed using the Python programming language (version 3.9.0)^30^ and Pytorch 1.11.0^31^. The imgaug library 0.4.0^26^ was used for data augmentation. We employed YOLOv7^32^ to develop a multiclass detection model for the six classified lesions. To identify the optimal hyperparameter configuration for achieving the best-performing model, we employed Hyperparameter Optimization with Genetic Algorithm for YOLOv7^32^ and presented the results as an optimized parameter table (Table 3). The hardware setup used for training included 2 * RTX 3090ti graphics processing units, 12th Generation Intel^®^ Core™ i9-12900K, and 32 GB RAM.

**Table 3:** Hyperparameters of the detection model after optimization with the genetic algorithm.

Hyperparameters	Default	Optimized hyperparameters
lr0	0.01	0.00096
lrf	0.1	0.127
Momentum	0.937	0.842
weight_decay	0.0005	0.00034
warmup_epochs	3.0	0.561 (fraction)
warmup_momentum	0.8	0.696
warmup_bias_lr	0.1	0.00986
iou_t	0.25	0.1
anchor_t	4.0	3.8

We also developed a classification model to distinguish the depth of invasion in cancer images. Based on the T stage from the pathological reports of resected specimens from patients with gastric cancer, we developed a binary classification model for T stage estimation.

Notably, we evaluated our model, especially on images showing discrepancies between the initial endoscopic impression and the actual T stage reported from the resected specimen. These included images from 13 patients who were initially thought to have EGC based on endoscopic findings but were upstaged to AGC after resection, and 75 patients who were initially thought to have AGC based on endoscopic findings but were downstaged to EGC after resection.

The primary outcome was lesion detection rate in the detection model. Additional performance metrics include the following:

The Positive Predictive Value (PPV), defined as “true positive / (true positive + false positive)”.

- Sensitivity, defined as “true positive / (true positive + false negative)”.

- Negative Predictive Value (NPV), defined as “true negative / (true negative + false negative)”.
- Specificity, defined as “true negative / (true negative + false positive)”.
- Accuracy, defined as “(true positive + true negative)/(true positive + true negative + false positive + false negative)”.

### Comparative performance analysis with experts

To compare the performance with experts, we conducted additional analysis using our AI model and four expert endoscopists. We collected an additional set of 104 anonymized endoscopic images, which were not part of our model's development dataset and were from a different period (2023–2024). These images were evenly distributed across six diagnostic classes. Both the model and four expert endoscopists independently reviewed these images without any prior knowledge of each other's assessments. Following their evaluations, we compiled and analyzed the results to assess and compare the diagnostic performance of the AI model and the experts.

### Ethics declarations

Approval of all ethical and experimental procedures and protocols was granted by the institutional review board (IRB) in Seoul National University Hospital (IRB No. 2108-030-1242). Due to the retrospective nature of the study, 2108-030-1242 waived the need of obtaining informed consent.

## Results

### Test performance of the computer-aided detection (CADe) model

**Figure 3:**
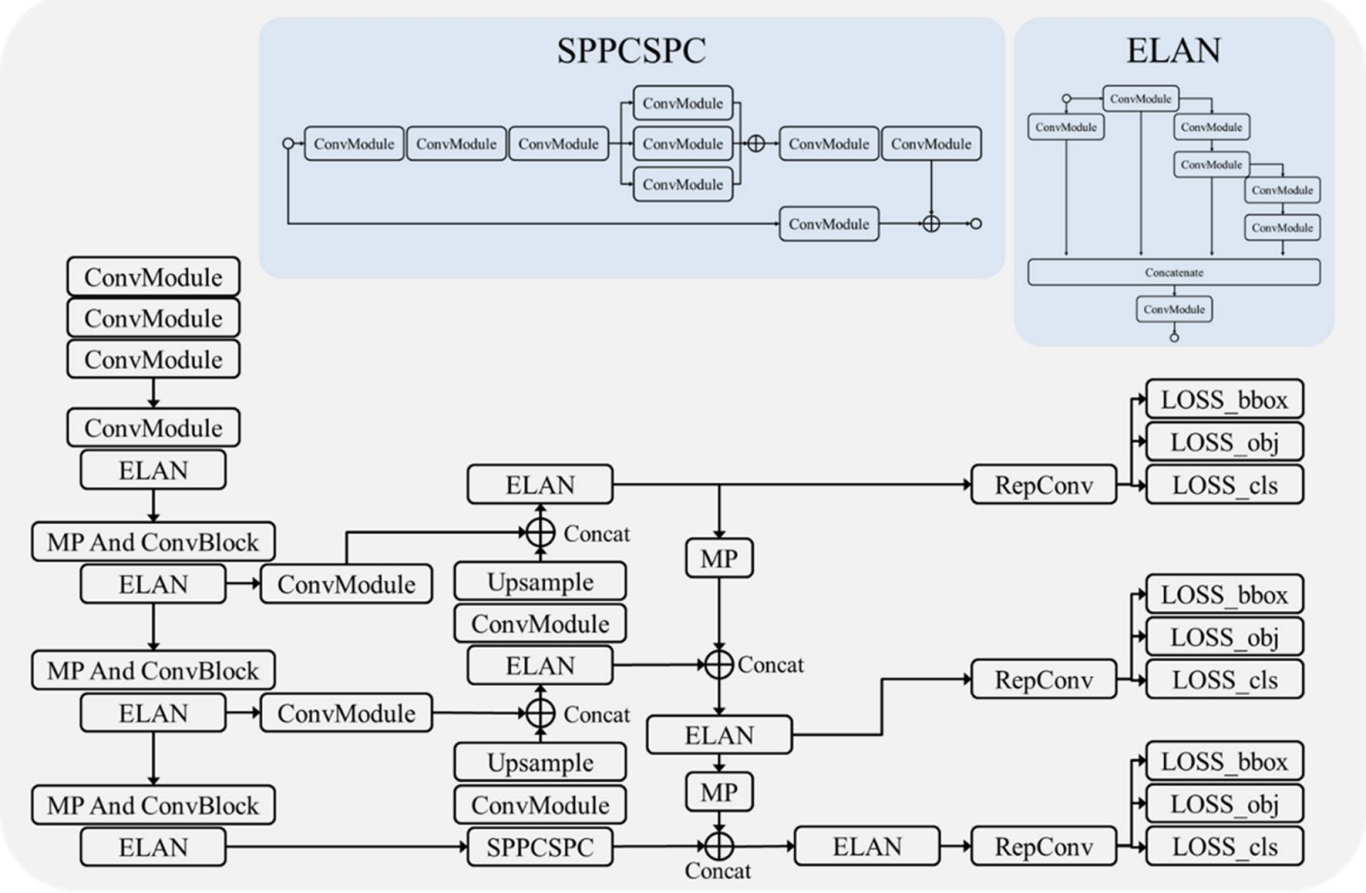
The structure of YOLOv7; MP: MaxPooling; ConvModule: Conv2d-BatchNormalization-SiLU activation; RepConv: Reparameterization.

A schematic of the established lesion detection system is depicted in Fig. 3. The lesion detection rate was assessed on a per-patient basis and achieved a rate of 95.22% (219 out of 230 patients) in the test set. In the context of endoscopic inspection, we opted to analyze the test results on a per-lesion basis, prioritizing continuous observation of specific lesions rather than relying on individual static image evaluations. This approach resulted in a 100% per lesion detection rate for EGC, 97.22% for AGC, 96.49% for dysplasia, 75.00% for BGU, 97.22% for benign polyps, and 80.49% for benign erosion. In internal testing of the classification models, the six-class category exhibited a maximum accuracy of 73.43% (95% CI 71.01–75.85%), accompanied by a sensitivity of 80.90% (95% CI 76.45–85.36%), specificity of 83.32% (95% CI 80.69–85.95%), PPV of 73.68% (95% CI 69.39–77.98%), and NPV of 88.53% (95% CI 86.29–90.77%) (Table 4). When categorized into cancer and non-cancer lesions, lesion detection rates were as high as 98.61% and 89.24%, respectively. Cancer lesions (EGC and AGC) demonstrated an NPV of 88.57% (95% CI 86.22–90.93%) and sensitivity of 78.62% (95% CI 72.39–84.85%). For neoplasms vs. non-neoplasms, the lesion detection rates were 97.67% and 85.15%, respectively. We also found high performance of CADe, achieving an 89.80% (95% CI 87.29–92.31%) NPV for neoplasms, indicating that it excels in detecting neoplastic lesions (Table 4).

**Table 4:** Diagnostic performance of the model in classifying lesions on endoscopic images.

Confidential threshold 0.25/ Iou Threshold 0.4	Negative predictive value, % (95% CI)	Positive predictive value, % (95% CI)	Specificity, % (95% CI)	Sensitivity, % (95% CI)	Accuracy, % (95% CI)
Six-class Classification	88.53 (86.29–90.77)	73.68 (69.39–77.98)	83.32 (80.69–85.95)	80.90 (76.45–85.36)	73.43 (71.01–75.85)
EGC	85.88 (80.31–91.45)	45.42 (35.97–54.86)	71.22 (66.33–76.10)	67.20 (54.56–79.85)	60.83 (56.83–64.83)
AGC	92.11 (88.59–95.63)	83.57 (71.42–95.73)	90.61 (84.61–96.61)	87.11 (81.48–92.75)	81.24 (74.08–88.40)
Dysplasia	92.06 (87.17–96.94)	85.14 (77.42–92.85)	89.92 (85.19–94.65)	87.47 (79.10–95.84)	80.68 (75.34–86.02)
BGU	87.87 (77.57–98.17)	56.79 (31.64–81.94)	91.01 (84.12–97.89)	56.25 (30.03–82.47)	60.95 (55.19–66.71)
Benign Polyp	94.09 (89.16–99.02)	79.54 (70.33–88.76)	82.54 (74.73–90.36)	92.24 (85.83–98.65)	85.44 (80.79–90.10)
Benign erosion	81.41 (74.04–88.78)	49.95 (31.03—68.87)	84.74 (77.55–91.92)	50.31 (29.33–71.29)	60.76 (54.25–67.28)
Neoplasm vs Benign					
Neoplasm	89.80 (87.29–92.31)	73.91 (69.02–78.80)	83.16 (80.52–85.80)	83.26 (78.51–88.01)	74.98 (71.83–78.13)
Non-neoplasm	86.23 (82.75–89.71)	74.08 (65.94–82.21)	84.60 (78.92–90.29)	75.17 (68.65–81.70)	70.22 (66.79–73.65)
Cancer vs Non-cancer					
Cancer	88.57 (86.22–90.93)	65.33 (56.01–74.64)	79.56 (75.22–83.90)	78.62 (72.39–84.85)	70.87 (66.62–75.12)
Non-Cancer	88.70 (85.33–92.07)	79.38 (73.64–85.13)	86.52 (82.40–90.64)	81.96 (76.00–87.91)	75.07 (71.95–78.19)
Cancer, depth of invasion					
T1	82.18%	87.04%	79.81%	88.68%	85.17%
≥ T2	87.04%	82.18%	88.68%	79.81%	85.17%

Cancer: AGC, EGC; Non-Cancer: dysplasia, BGU, benign polyp, benign erosion; Neoplasm: AGC, EGC, dysplasia; Non-Neoplasm: BGU, benign polyp, benign erosion.

The detailed training parameters for the lesion detection model were as follows:

- Augmentation methods: Horizontal flip, HSV channel translation, translation, scale, mosaic, and copy-paste.
- Batch size: 64
- Epoch: 100
- Optimizer: SGD with a momentum of 0.842
- Learning rate scheduler OneCycleLR
- Label smoothing: 0.1

### Test performance of the T-stage classification model

In this study, we developed an algorithm to classify cancer images and their depth of invasion into T stages. For this purpose, we employ the EfficientNet-B3 model. When the T stage was classified as T1 and T2-T4, the model achieved an accuracy of 85.17%, sensitivity of 88.68%, specificity of 79.81%, PPV of 87.04%, and NPV of 82.18% (Table 4). Notably, among 75 pathologically proven patients with AGC, with the initial impression of the endoscopist being EGC, our model accurately predicted the T stage in 65 patients. In addition, out of 13 patients diagnosed by their endoscopist as having AGC,, their` actual T stage was EGC, and the model accurately predicted the T stage in nine cases.

The detailed training parameters for the lesion detection model were as follows:

- Batch size: 48
- Epoch: 100
- Optimizer: SGD with momentum of 0.9, decay of 0.00034
- Learning rate: 0.0096
- Loss function: sparse categorical cross-entropy

### Comparative performance analysis with experts

In our expanded analysis involving both four expert endoscopists and our CNN model, we found that our model performed robustly across a diverse dataset of six lesion types. We analyzed our model's performance across various lesion types including the performance to distinguish between cancer and non-cancer, neoplasm and non-neoplasm, each 6 lesion types (Supplementary Table 1–3). In the classification between cancer versus non-cancer, which is the most important issue in endoscopic exam, the negative predictive value and sensitivity of the AI was 88.89% and 98.51% which was superior than that of experts (78.38% and 88.06% respectively) (Supplementary Table 1).

**Figure 4:**
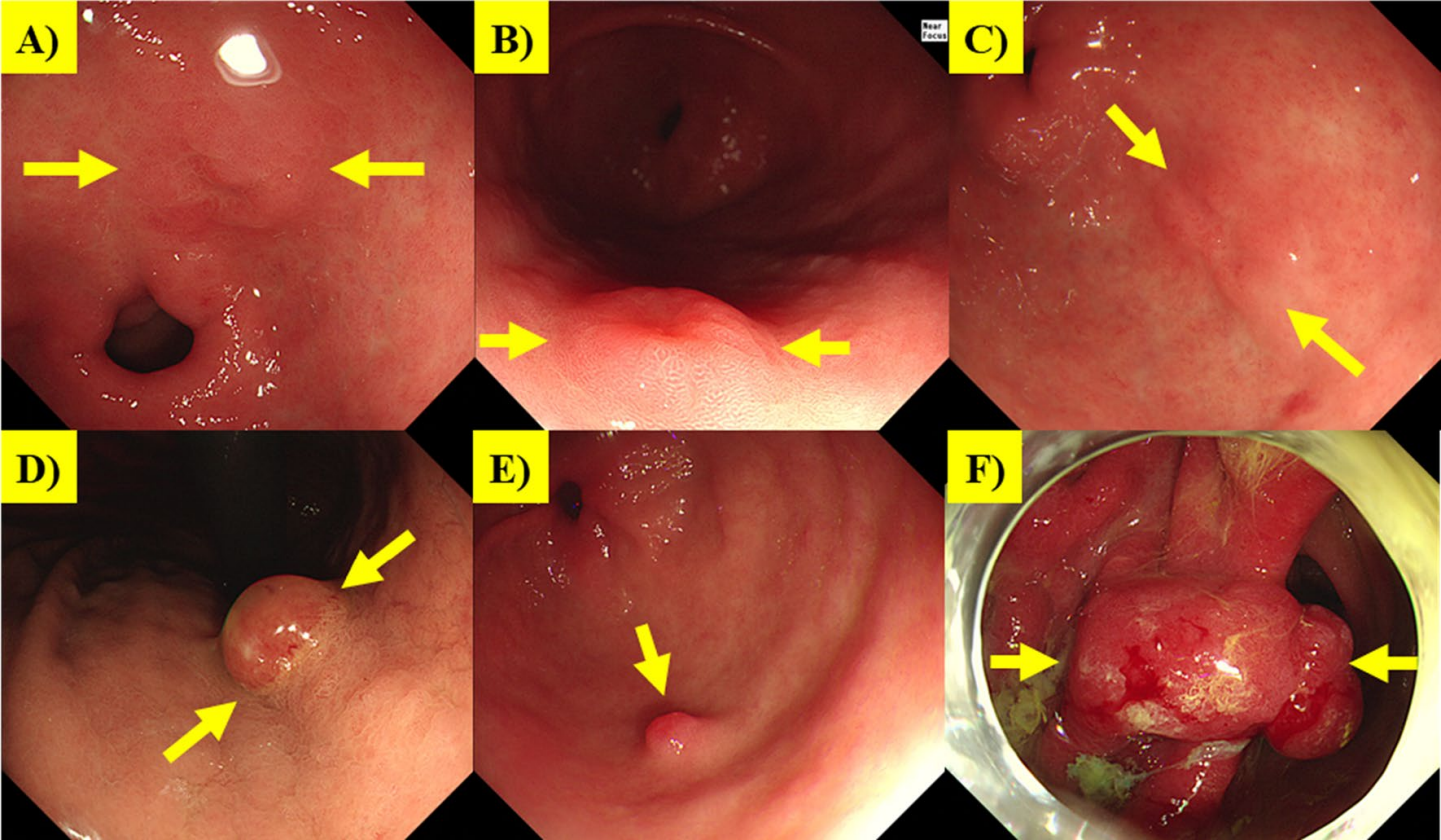
Representative cases where the AI correctly classified the lesion, while experts did not. (**A**, **B**) Cases where AI accurately recognized Dysplasia, while experts categorized the same lesions as Benign erosion. (**C**) Cases where AI accurately recognized Dysplasia, while experts categorized the same lesions as EGC. (**D**, **E**) Cases where AI accurately recognized Benign Polyps while misclassified as dysplasia. (**F**) Cases where AI accurately recognized Benign Polyps while misclassified as AGC.

We further identified every case where our model correctly classified lesion types, while expert endoscopists did not (Supplementary Table 4) with representative images (Fig. 4). Our model accurately recognized dysplasia, in cases where some experts categorized the same lesions as benign erosion (Fig. 4A, B) or EGC (Fig. 4C). Furthermore, our model successfully identified benign polyps in cases where they were misclassified as dysplasia (Fig. 4D, E) or AGC (Fig. 4F) by some experts. This is of particular importance as such distinctions can have significant implications on subsequent clinical management, including treatment decisions and follow-up endoscopy scheduling.

## Discussion

In this study, we developed an automated system for detecting and classifying malignant, premalignant, and benign gastric lesions. Previous studies^25^ aimed to classify lesions using a separate artificial intelligence model for images detected using an anomaly detection algorithm. However, this approach requires an additional step to indicate the required histological examination, even after lesion detection, deviating from the primary goal of reducing the workload and fatigue^23–25^. To overcome this limitation, we developed a multiclass detection algorithm with real-time processing to eliminate the need for dual processing. Although detection algorithms with high sensitivity can identify subtle lesions, there is a risk of overprediction. Misclassifying nonlesions as lesions can inundate clinicians with false alarms, possibly overshadowing true lesions and misguiding lesion classification^9,10^. This can compound clinician fatigue, which is a challenge that CADe aims to address. In addition, a major concern during endoscopic examinations is the possibility of missed lesions such as cancers. Considering these challenges, our algorithm was primarily tailored to yield a high NPV for cancerous and neoplastic lesions^11^. From the data encompassing 2,606 patients, we achieved an NPV of 88.53% and a sensitivity of 80.90% for the six-class classification. Notably, cancerous lesions had an NPV of 88.57% and sensitivity of 78.62%, and neoplastic lesions had an NPV of 89.80% and sensitivity of 83.26%. Overall, the detection rate was 95.22% in the 306 patients evaluated.

The decision to perform a biopsy is often based on the assessment of the malignant and premalignant potential of a lesion. This nuanced judgment is based on extensive endoscopic experience. Our methodology can substantially aid in refining biopsy decisions and in classifying lesions as cancerous, neoplastic, or benign.

In addition, when estimating the depth of invasion for gastric cancer, discrepancies between endoscopist impressions and the actual pathological T stage are often observed. Our model achieved high performance in the prediction of the T stage, even in cases that showed discrepancies in the endoscopic and pathological T stages. Because treatment options for gastric cancer, such as endoscopic or surgical resection, often rely on the estimated T stage, this model could aid in accurate decisions for optimal treatment.

However, this study has a few limitations. The data used in this study were exclusively obtained from a single institution. Therefore, the algorithms employed in this study require external validation. Moreover, owing to the nature of Seoul National University Hospital, which is a tertiary hospital, there was a higher proportion of advanced cancer cases than of EGC or BGU cases, resulting in somewhat lower evaluation metrics, especially in the BGU and EGC classes. Additionally, data imbalance raises the possibility of bias towards cancerous lesions. Hence, securing multi-institutional data is recommended for external validation in future studies. Furthermore, because we could not compare the performance of the developed algorithm with that of an endoscopist using the same images, it is impossible to determine the true extent of the algorithm’s enhancement of the lesion detection rate or its potential impact.

Compared with previous studies^12,16,22,23^, the strength of this study is its comprehensive inclusion of various lesions. Not only did we account for diverse cancers and premalignant lesions, but we also incorporated common benign lesions that show diverse endoscopic appearances, including BGUs, benign polyps, and benign erosions. This wide-ranging analysis ensured a thorough and representative evaluation, thus enhancing the applicability and robustness of the findings.

In summary, our model, which was tailored for detecting and classifying gastric cancers, dysplasia, and various benign lesions, demonstrated an outstanding performance and has the potential to assist clinicians in decision-making during endoscopic procedures.

### Supplementary Information

Supplementary Information.

## Data Availability

The data generated and/or analyzed during the current study are available from the corresponding author on reasonable request.
