# Supplementary material for: Enhanced multi-class pathology lesion detection in gastric neoplasms using deep learning-based approach and validation
Source: Sci Rep. 2024 May 21;14:11527. doi: 10.1038/s41598-024-62494-1 (PMC11109266; doi:10.1038/s41598-024-62494-1)
Supplement: Supplementary file 1 — Supplementary Information. [file 41598_2024_62494_MOESM1_ESM.docx]

**Supplementary information**

**Enhanced Multi-Class Pathology Lesion Detection in Gastric Neoplasms using Deep Learning-Based Approach and Validation**

Byeong Soo Kim^1, †^, Bokyung Kim^2, †^, Minwoo Cho^3^, Hyunsoo Chung^4^, Ji Kon Ryu^4, *^, and Sungwan Kim^5, 6, *^

^1^Interdisciplinary Program in Bioengineering, Graduate School, Seoul National University, Seoul, 08826, Korea

^2^Division of Gastroenterology, Department of Internal Medicine Seoul Metropolitan Government Seoul National University Boramae Medical Center, Seoul 07061, Korea

^3^Transdisciplinary Department of Medicine, Seoul National University Hospital, Seoul, 03080, Korea

^4^Department of Internal Medicine and Liver Research Institute, Seoul National University Hospital, Seoul National University College of Medicine, Seoul, 03080, Korea

^5^Department of Biomedical Engineering, Seoul National University College of Medicine, Seoul, 03080, Korea

^6^Artificial Intelligence Institute, Seoul National University, Seoul, 08826, Korea

^*^ jkryu@snu.ac.kr (J.K. Ryu); [sungwan@snu.ac.kr](mailto:sungwan@snu.ac.kr) (S. Kim)

^†^ Byeong Soo Kim and Bokyung Kim contributed equally to this article.

**Ji Kon Ryu**

Department of Internal Medicine and Liver Research Institute, Seoul National University Hospital, Seoul National University College of Medicine, Seoul, 03080, Korea

jkryu@snu.ac.kr

**Sungwan Kim**

Department of Biomedical Engineering, Seoul National University College of Medicine, 101 Daehak-ro, Jongno-gu, Seoul 03080, Korea

Tel: +82-2-2072-3126

Fax: +82-2-745-7870

E-mail: sungwan@snu.ac.kr

**Supplementary Table 1. Performance of the proposed model and experts in diagnosing cancer (EGC and AGC) versus non-cancer lesions**

| **Metrics** | **Our detection model (95% CI)** | **Experts (n = 4) (95% CI)** |
| --- | --- | --- |
| Accuracy | 75.51% (65.79%, 83.64%) | 89.80% (82.03%, 95.00%) |
| Sensitivity | 98.51% (92.82%, 99.75%) | 88.06% (79.59%, 93.51%) |
| Specificity | 25.81% (17.24%, 35.31%) | 93.55% (85.84%, 97.08%) |
| PPV | 74.16% (63.59%, 81.88%) | 96.72% (89.88%, 98.88%) |
| NPV | 88.89% (80.80%, 94.26%) | 78.38% (68.01%, 85.36%) |

PPV, positive predictive value; NPV, negative predictive value

**Supplementary Table 2. Performance of the proposed model and experts in diagnosing neoplasms (Dysplasia, EGC, and AGC) versus non-neoplastic lesions**

| **Metrics** | **Our detection model (95% CI)** | **Experts (n = 4) (95% CI)** |
| --- | --- | --- |
| Accuracy | 78.85% (70.04%, 85.59%) | 86.30% (82.66%, 89.27%) |
| Sensitivity | 85.45% (77.42%, 90.96%) | 90.45% (87.25%, 92.92%) |
| Specificity | 71.43% (62.10%, 79.23%) | 81.63% (77.63%, 85.06%) |
| PPV | 77.05% (68.09%, 84.08%) | 85.09% (81.35%, 88.19%) |
| NPV | 81.40% (72.85%, 87.71%) | 88.14% (84.68%, 90.90%) |

EGC, early gastric cancer; AGC, advanced gastric cancer; PPV, positive predictive value; NPV, negative predictive value

**Supplementary Table 3. Performance of the proposed model and experts in the six-class classification of lesions**

| **Lesion Type** | **Metrics** | **Our detection model (95% CI)** | **Experts (n = 4) (95% CI)** |
| --- | --- | --- | --- |
| BGU | Accuracy | 95.19% (89.24% - 97.93%) | 93.33% (90.74% - 95.24%) |
|  | Sensitivity | 16.67% (3.01% - 56.35%) | 68.75% (57.93% - 77.85%) |
|  | Specificity | 100.00% (96.23% - 100.00%) | 98.25% (96.43% - 99.15%) |
|  | PPV | 100.00% (20.65% - 100.00%) | 88.04% (77.67% - 93.97%) |
|  | NPV | 95.15% (89.14% - 97.91%) | 94.04% (91.35% - 95.94%) |
| Benign erosions | Accuracy | 80.77% (72.15% - 87.19%) | 93.12% (90.50% - 95.06%) |
|  | Sensitivity | 56.00% (37.07% - 73.33%) | 80.00% (69.95% - 87.30%) |
|  | Specificity | 88.61% (79.75% - 93.89%) | 95.75% (93.30% - 97.33%) |
|  | PPV | 60.87% (40.79% - 77.84%) | 79.17% (69.11% - 86.59%) |
|  | NPV | 86.42% (77.30% - 92.24%) | 96.02% (93.63% - 97.54%) |
| Benign polyps | Accuracy | 97.12% (91.86% - 99.01%) | 97.50% (95.68% - 98.56%) |
|  | Sensitivity | 90.00% (69.90% - 97.21%) | 88.75% (79.98% - 93.97%) |
|  | Specificity | 98.81% (93.56% - 99.79%) | 99.25% (97.82% - 99.74%) |
|  | PPV | 94.74% (75.36% - 99.06%) | 95.75% (88.48% - 98.51%) |
|  | NPV | 97.65% (91.82% - 99.35%) | 97.84% (95.91% - 98.87%) |
| Dysplasia | Accuracy | 62.50% (52.91% - 71.20%) | 88.96% (85.84% - 91.46%) |
|  | Sensitivity | 92.31% (66.69% - 98.63%) | 63.75% (52.81% - 73.43%) |
|  | Specificity | 58.24% (47.98% - 67.84%) | 94.00% (91.23% - 95.94%) |
|  | PPV | 24.00% (14.30% - 37.41%) | 72.53% (61.52% - 81.35%) |
|  | NPV | 98.15% (90.23% - 99.67%) | 92.82% (89.89% - 94.95%) |
| EGC | Accuracy | 78.85% (70.04% - 85.59%) | 84.38% (80.86% - 87.35%) |
|  | Sensitivity | 0.00% (0.00% - 14.87%) | 72.50% (61.86% - 81.08%) |
|  | Specificity | 100.00% (95.52% - 100.00%) | 86.75% (83.08% - 89.73%) |
|  | PPV | 0.00% (0.00% - 0.00%) | 52.81% (43.58% - 61.84%) |
|  | NPV | 78.85% (70.04% - 85.59%) | 94.01% (91.10% - 96.00%) |
| AGC | Accuracy | 93.27% (86.75% - 96.70%) | 93.96% (91.46% - 95.76%) |
|  | Sensitivity | 61.11% (38.62% - 79.69%) | 80.00% (69.95% - 87.30%) |
|  | Specificity | 100.00% (95.72% - 100.00%) | 96.75% (94.52% - 98.09%) |
|  | PPV | 100.00% (74.12% - 100.00%) | 83.59% (73.76% - 90.22%) |
|  | NPV | 92.47% (85.27% - 96.31%) | 96.09% (93.72% - 97.58%) |

EGC, early gastric cancer; AGC, advanced gastric cancer; PPV, positive predictive value; NPV, negative predictive value

**Supplementary Table 4. Cases in which the proposed model correctly classified the lesion, while experts did not.**

| Lesion (Ground truth) | Our detection model | Expert 1 | Expert 2 | Expert 3 | Expert 4 |
| --- | --- | --- | --- | --- | --- |
| AGC | AGC | EGC | AGC | AGC | AGC |
| AGC | AGC | EGC | AGC | AGC | AGC |
| AGC | AGC | AGC | AGC | BGU | AGC |
| Dysplasia | Dysplasia | Benign erosion | Benign erosion | Benign erosion | Dysplasia |
| Dysplasia | Dysplasia | Benign erosion | Benign erosion | Benign erosion | Dysplasia |
| Dysplasia | Dysplasia | Dysplasia | Benign erosion | Benign erosion | Dysplasia |
| Dysplasia | Dysplasia | Dysplasia | EGC | Benign erosion | Benign erosion |
| Dysplasia | Dysplasia | Dysplasia | EGC | Dysplasia | Dysplasia |
| Dysplasia | Dysplasia | EGC | EGC | EGC | EGC |
| Dysplasia | Dysplasia | Dysplasia | EGC | EGC | Dysplasia |
| Dysplasia | Dysplasia | Dysplasia | EGC | EGC | EGC |
| Dysplasia | Dysplasia | Benign erosion | Dysplasia | EGC | EGC |
| Benign polyp | Benign polyp | AGC | Benign polyp | AGC | Benign polyp |
| Benign polyp | Benign polyp | Dysplasia | Benign polyp | Benign polyp | Benign polyp |
| Benign polyp | Benign polyp | Dysplasia | Benign polyp | Benign polyp | Benign polyp |
| Benign polyp | Benign polyp | Benign polyp | Benign polyp | Dysplasia | Benign polyp |
| Benign polyp | Benign polyp | Benign polyp | Benign polyp | Dysplasia | Benign polyp |
| Benign polyp | Benign polyp | Benign polyp | Benign polyp | Dysplasia | Benign polyp |
| Benign polyp | Benign polyp | Benign polyp | Benign polyp | EGC | Benign polyp |
| Benign polyp | Benign polyp | Benign polyp | Benign polyp | EGC | Benign polyp |
| Benign erosion | Benign erosion | Benign erosion | Benign erosion | BGU | Benign erosion |
| Benign erosion | Benign erosion | Dysplasia | Benign erosion | Dysplasia | Benign erosion |

EGC, early gastric cancer; AGC, advanced gastric cancer; BGU, benign gastric ulcer
